# 

**Published:** 1897-08

**Authors:** 


					﻿Southern Medical Record.
A Monthly Journal of Medicine and Surgery.
^ol. XXVII.- ATLANTA, GA., AUGUST, 1897.	No. 8^
BERNARD WOLFF, M. D., Editor.
Associate Editors:
A. W. GRIGGS, M. D.	WILLIS F. WESTMORELAND, M. D.
j. mcfadden gaston, m. d.
E. Y. CLARKE, Genebal Manager.	B. M. ZETTLER, Business Manager.
Entered at the Post-office at Atlanta, Ga., as Second-class Matter.
The Foote & Davies Co., Atlanta, Ga.
				

